# Genomic Profiling Identifies Putative Pathogenic Alterations in NSCLC Brain Metastases

**DOI:** 10.1016/j.jtocrr.2022.100435

**Published:** 2022-11-11

**Authors:** Marcin Nicoś, Luuk Harbers, Enrico Patrucco, Maximilian Kramer-Drauberg, Xiaolu Zhang, Claudia Voena, Anna Kowalczyk, Aleksandra Bożyk, Rafał Pęksa, Bożena Jarosz, Justyna Szumiło, Michele Simonetti, Monika Żuk, Bartosz Wasąg, Katarzyna Reszka, Renata Duchnowska, Janusz Milanowski, Roberto Chiarle, Magda Bienko, Paweł Krawczyk, Jacek Jassem, Chiara Ambrogio, Nicola Crosetto

**Affiliations:** a Department of Microbiology, Tumor and Cell Biology, Karolinska Institutet, Stockholm, Sweden; b Science for Life Laboratory, Solna, Sweden; c Department of Pneumonology, Oncology and Allergology, Medical University of Lublin, Lublin, Poland; d Department of Molecular Biotechnology and Health Sciences, University of Torino, Torino, Italy; e Current address: Department of Physiology and Pathophysiology, School of Basic Medicine, Shandong University, Jinan, People’s Republic of China; f Department of Oncology and Radiotherapy, Medical University of Gdansk, Gdańsk, Poland; g Polish Brain Metastases Consortium, Central and East European Oncology Group, Gdańsk, Poland; h Department of Pathology, Medical University of Gdansk, Gdańsk, Poland; i Department of Neurosurgery and Pediatric Neurosurgery, Medical University of Lublin, Lublin, Poland; j Department of Pathomorphology, Medical University of Lublin, Lublin, Poland; k Department of Biology and Medical Genetics, Medical University of Gdansk, Gdańsk, Poland; l Genetics and Immunology Institute of Lublin, Genim LLC, Lublin, Poland; m Department of Oncology, Military Institute in Warsaw, Warsaw, Poland; n Department of Pathology, Boston Children’s Hospital and Harvard Medical School, Boston, Massachusetts; o Genomics Research Centre, Human Technopole, Milan, Italy

**Keywords:** Non–small cell lung cancer (NSCLC), Brain metastases, Genomic profiling, Targetable pathogenic alterations

## Abstract

**Introduction:**

Brain metastases (BM) severely affect the prognosis and quality of life of patients with NSCLC. Recently, molecularly targeted agents were found to have promising activity against BM in patients with NSCLC whose primary tumors carry “druggable” mutations. Nevertheless, it remains critical to identify specific pathogenic alterations that drive NSCLC-BM and that can provide novel and more effective therapeutic targets.

**Methods:**

To identify potentially targetable pathogenic alterations in NSCLC-BM, we profiled somatic copy number alterations (SCNAs) in 51 matched pairs of primary NSCLC and BM samples from 33 patients with lung adenocarcinoma and 18 patients with lung squamous cell carcinoma. In addition, we performed multiregion copy number profiling on 15 BM samples and whole-exome sequencing on 40 of 51 NSCLC-BM pairs.

**Results:**

BM consistently had a higher burden of SCNAs compared with the matched primary tumors, and SCNAs were typically homogeneously distributed within BM, suggesting BM do not undergo extensive evolution once formed. By comparing focal SCNAs in matched NSCLC-BM pairs, we identified putative BM-driving alterations affecting multiple cancer genes, including several potentially targetable alterations in genes such as *CDK12*, *DDR2*, *ERBB2*, and *NTRK1*, which we validated in an independent cohort of 84 BM samples. Finally, we identified putative pathogenic alterations in multiple cancer genes, including genes involved in epigenome editing and 3D genome organization, such as *EP300*, *CTCF*, and *STAG2*, which we validated by targeted sequencing of an independent cohort of 115 BM samples.

**Conclusions:**

Our study represents the most comprehensive genomic characterization of NSCLC-BM available to date, paving the way to functional studies aimed at assessing the potential of the identified pathogenic alterations as clinical biomarkers and targets.

## Introduction

Brain metastases (BM) are detected in 20% to 40% of patients with NSCLC at the time of diagnosis, and eventually 50% of the patients succumb to them.^1^ Owing to limited blood-brain barrier permeability,^2^^,^^3^ NSCLC-BM present lower sensitivity to most cytotoxic and immune agents compared with extracranial sites.^4^^,^^5^ Next-generation *EGFR* and *ALK* inhibitors and *ROS1*, *RET*, *MET*, and *NTRK* inhibitors that penetrate the blood-brain barrier were found to have promising activity against BM in patients with NSCLC whose primary tumors carry mutations in these genes.^6^^,^^7^ Nevertheless, it remains critical to identify specific pathogenic alterations in NSCLC-BM which can provide novel and more effective therapeutic targets.

Several studies have mapped the genomic landscape of BM in general and, specifically, of NSCLC-BM, aiming at identifying potential pathogenic alterations driving BM formation or progression. In one study, whole-exome sequencing (WES) and RNA sequencing were performed in 36 BM of various origins, including 18 NSCLC-BM samples, identifying potentially actionable alterations in several genes including *AKT1*, *CDK6*, *EGFR*, *MEK1*, and *MET*.^8^ In a large-scale pan-cancer study, 2583 metastases from 20 different tumor types were profiled by whole-genome sequencing (WGS).^9^ Nevertheless, only one sample was a NSCLC-BM.^9^ In another study, 73 pairs of lung cancer adenocarcinoma (LUAD) and matched BM were profiled by WES and low-pass WGS for calling somatic copy number alterations (SCNAs), leading to the identification of *MYC*, *YAP1*, and *MMP13* amplifications and *CDKN2A/B* deletions as pathogenic alterations in LUAD-BM.^10^ More recently, 12 pairs of primary NSCLC and matched BM were profiled by WES, revealing several BM-associated mutations in known cancer genes, including *AHNAK2*, *ANKRD36C*, *BAGE2*, *KMT2C*, and *PDE4DIP*.^11^ In another study, WES profiling of 10 pairs of primary LUAD samples and matched BM and liver metastases revealed distinct metastatic mutational landscapes and evolutionary patterns.^12^ NSCLC-BM have also been profiled by targeted gene sequencing in the attempt to identify potentially actionable BM-driving mutations. In one study, 416 genes were profiled in 61 matched primary NSCLC and BM samples, revealing a correlation between PI3K pathway mutations and increased risk of BM.^13^ In another study, sequencing of 315 genes in 85 nonpaired NSCLC and BM samples revealed a worse prognosis for patients carrying LUAD-BM with *CREBBP*, *GPR124*, or *SPTA1* mutations.^14^ Altogether, these studies have begun casting light on the genomic architecture of NSCLC-BM and uncovered potentially actionable pathogenic alterations in NSCLC-BM. Nevertheless, further comparative genomic studies are needed to comprehensively chart the genomic landscape of BM in the two main NSCLC histologic subtypes (LUAD and lung squamous cellular carcinoma [LUSC]) and in different populations, to identify actionable pathogenic alterations specific to BM which can be tested in follow-up studies.

To this end, we applied shallow WGS to perform a comparative genomic characterization of primary NSCLC and matched BM samples aimed at uncovering potentially targetable alterations that could be used to design novel precision treatment strategies for patients with NSCLC. We profiled SCNAs in 51 matched pairs of primary NSCLC and BM samples and validated the identified potentially pathogenic BM-driving alterations in an independent cohort of 84 BM samples. Next, we assessed the SCNA spatial distribution within BM by performing multiregion SCNA profiling in 15 BM samples. Finally, we performed WES on 40 NSCLC-BM pairs to identify pathogenic mutations specific to BM, which we validated by targeted sequencing of an independent cohort of 115 BM samples. Our study represents the most comprehensive genomic characterization of LUAD and LUSC BM available to date.

## Materials and Methods

### Experimental Methods

#### Samples

The collection of all tumor samples described in this study was approved by the Ethics Committee of the Medical University of Lublin, Poland, under ethical permit no. KE-0254/235/2016. All samples were collected between 2005 and 2015.

##### Discovery Cohort

We retrieved 51 pairs of archival, formalin-fixed, paraffin-embedded (FFPE) NSCLC primary tumor and matched BM samples, including 33 pairs from patients diagnosed with having LUAD and 18 from patients diagnosed with having LUSC. The samples included surgical and diagnostic (biopsy) specimens that had been previously collected at the Medical University of Lublin (Poland) and at the Medical University of Gdansk (Poland). Data on identified oncogenic drivers in the primary lesions were available in 44 patients, with 17 of 44 (38.6%) tumor samples containing oncogenic *KRAS* mutations and six of 44 (13.6%) samples containing *EGFR* gene substitutions. One patient (2.3%) had a single rearrangement of *ALK* in the primary tumor and another patient (2.3%) had an *NTRK1* rearrangement. Furthermore, of 44 patients, 19 (43.2%) were considered wild type in terms of the other genes covered by the FusionPlex Lung panel (ArcherDX, Boulder, CO).^15^ At the time of biopsy or surgical resection of the primary tumor, all patients were naive to chemo-, radio-, immune-, and molecularly targeted therapies. The BM samples were obtained during neurosurgery, and the median time between the primary tumor diagnosis and the detection of the corresponding BM was 12 plus and minus 17 months (mean ± SD, range: 1–78 mo). We defined BM as synchronous (n = 12) if they were detected between 0 and 2 months; early (n = 14) if they were detected between 3 and 12 months; and late (n = 25) if they were detected more than 12 months since the primary diagnosis of lung cancer. The median age of the patients at the moment of diagnosis was 60 plus and minus 8 years (mean ± SD, range 39–79 y). In addition, 25 patients were above or equal to 60 years old and 26 were less than 60 years old. There were 18 female and 33 male patients. Furthermore, of the patients, 37 were smokers and 14 nonsmokers, according to their smoking habits at the time of diagnosis. The median overall survival (mOS) from the time of the primary tumor diagnosis was 26 plus and minus 31 months (range 2–184 mo), whereas the mOS from the time of BM diagnosis was shorter (7 ± 26 mo, mean ± SD; range 1–160 mo).

##### Validation Cohort

We retrieved archival FFPE tissue sections from 115 NSCLC-BM surgically resected at the same institutions where the discovery cohort samples were collected. Because only fine-needle aspiration biopsies were performed on the primary tumors, we could not obtain enough genomic DNA (gDNA) to genomically profile both primary tumors and matched BM. There were 83 patients diagnosed with having LUAD and 32 with having LUSC. The median age of these patients was 66 plus and minus 8 years (mean ± SD, range 44–86 y). There were 37 patients above or equal to 60 years old and 78 patients less than 60 years old. Of the patients, 76 were females and 39 males. Information about smoking status, mOS, and median time to BM development was not available.

#### gDNA Extraction and Sonication

We cut two consecutive sections (4 μm and 8 μm thick, respectively) from each FFPE tissue block in the discovery and validation cohorts and used the 4-μm-thick section for hematoxylin and eosin staining and the 8-μm-thick section for gDNA extraction. To extract gDNA, we used the QIAamp DNA FFPE Tissue Kit (Qiagen, Germany) following the manufacturer’s protocol. We assessed the quality and quantity of the gDNA samples using a NanoDrop 2000 and a Qubit 3.0 fluorimeter (Thermo Fisher Scientific, Germany) and retained only samples that had a range of light absorption (A_260_/A_280_) comprised between 1.8 and 2.0. We sheared 6 to 10 ng of each purified gDNA using a Bioraptor Plus (Diagenode, Germany) with cycling conditions optimized to achieve a mean target size of 150 to 200 base pairs (bp). We evaluated the distribution of the sheared gDNA on a Bioanalyzer 2100 (Agilent Technologies, UK) using a High Sensitivity DNA Kit. We remeasured the quantity of the sheared gDNA on a Qubit fluorometer and stored the samples at −20°C until we prepared sequencing libraries.

#### SCNA Profiling in Matched NSCLC Primary and BM Samples

To profile SCNAs in the discovery cohort, we prepared individual sequencing libraries from each of the 51 pairs of NSCLC and BM samples in the cohort using the NEBNext Ultra II DNA Library Prep Kit for Illumina and corresponding NEBNext Multiplex Oligos for Illumina (New England Biolabs, UK) following the manufacturer’s instructions. We assessed the size distribution, quality, and quantity of each library on a Bioanalyzer 2100 and sequenced the libraries shallowly on a NextSeq 500 system (Illumina, San Diego, CA) using a NextSeq 500/550 High Output v2 kit (75 cycles).

#### SCNA Validation on NanoString

To validate SCNAs identified in the discovery cohort, we applied the nCounter v2 Cancer CN Assay (NanoString, Seattle, WA) to four NSCLC-BM pairs and one additional BM sample for which we had enough (150–200 ng) gDNA. We requantified the gDNA concentration of each sample and then handed it to the Karolinska Institutet Gene facility (Stockholm, Sweden) for data collection. We analyzed the resulting data using the freely available nCounter Analysis Software.

#### Multiregion SCNA Profiling

To assess how the SCNAs identified in the discovery cohort were spatially distributed in the BM samples, we used the CUTseq method that we previously developed and applied to study the spatial heterogeneity of SCNAs in breast cancer FFPE samples.^16^ Owing to the scarcity of the neurosurgical material available, we only managed to retrieve individual FFPE tissue sections from 15 BM samples containing two to four clearly separated (n = 5 sections) or sufficiently large (n = 10 sections) lesions so that we were able to extract DNA from two to four separated circular regions of similar size (approximately 1 cm^2^). To extract gDNA from each region, we applied the PinPoint Solution (Zymo Research, Irvine, CA) onto each region, and after 30 minutes, we scraped the solidified solution and performed gDNA extraction and purification using a standard phenol-chloroform protocol followed by quantification of the samples using a Qubit 3.0 fluorimeter. We applied CUTseq^16^ to prepare multiplexed sequencing libraries by pooling the gDNA extracted from up to 71 different regions together. We assessed the size distribution, quality, and quantity of the libraries on a Bioanalyzer 2100 using a High Sensitivity DNA kit and sequenced them on a NextSeq 500 system using a NextSeq 500/550 High Output v2 kit (75 cycles).

#### Validation of Putative BM-Driving SCNAs

To validate the putative pathogenic SCNAs identified in the discovery cohort, we applied CUTseq^16^ to 84 BM samples in the validation cohort, for which we had enough gDNA. We performed CUTseq in 96-well plates using a low-volume contactless liquid-dispensing device (I.DOT One, Dispendix GmbH, Germany) to dispense the CUTseq digestion and ligation mix. We then pooled the samples (24 samples per pool) before performing in vitro transcription and preparing a multiplexed sequencing library from each pool using the CUTseq protocol. We assessed the quality of each library using a Bioanalyzer 2100 and sequenced all libraries on a NextSeq 500 system using a NextSeq 500/550 High Output v2 kit (75 cycles).

#### WES and Validation of Putative BM-Driving Mutations

To identify putative pathogenic mutations that might be driving BM, we performed WES in 40 NSCLC primary and BM sample pairs in the discovery cohort, for which we had enough gDNA. We first prepared a sequencing library from each sample using the SureSelect XT HS Kit (Agilent Technologies, UK) following the manufacturer’s instructions and assessed the size distribution, quality, and quantity of each library on a Bioanalyzer 2100. To reach the recommended input for exome capture (500–1500 ng), we pooled up to eight libraries together and concentrated the pools using the Savant SpeedVac DNA 130 Integrated Vacuum Concentrator System (Thermo Fisher Scientific, Germany) using the standard heating mode, until all the solutions were entirely evaporated. We resuspended each pool in 12 μL of nuclease-free water and performed exome capture using the SureSelect XT HS Target Enrichment kit and SureSelect Human All Exon v6 baits (Agilent Technologies, UK) following the manufacturer’s protocol. We again assessed the size distribution, quality, and quantity of all captured libraries on a Bioanalyzer 2100 and Qubit and sequenced all libraries on a NovaSeq 6000 system (Illumina, San Diego, CA) at the National Genomics Infrastructure (NGI Stockholm, Sweden) using a 2 × 150 bp flowcell S4 (Illumina, San Diego, CA).

To validate the putative pathogenic mutations identified in the discovery cohort, we performed targeted gene sequencing of all the 115 BM samples in the validation cohort using the SureSelect CD Glasgow Cancer Core Panel (Agilent Technologies, UK), which captures 174 cancer-associated genes, following the manufacturer’s instructions. We again assessed the size distribution, quality, and quantity of all captured libraries using Bioanalyzer 2100 and Qubit and then sequenced all libraries on a NovaSeq 6000 platform at the NGI (Stockholm, Sweden) using a 2 × 150 bp flowcell S4.

### Computational Methods

#### Sequencing Data Processing

We demultiplexed all raw sequence reads to FASTQ files using the BaseSpace Sequence Hub cloud service of Illumina. For CUTseq data, we further demultiplexed the reads to sample specific FASTQ files using a custom Python script available at https://github.com/ljwharbers/metastatic_lungcancer. Next, we aligned all reads to the GRCh37/hg19 reference genome using *BWA* (v0.7.17-r1188)^17^ and then sorted and indexed them using *SAMtools* (v1.10).^18^ For CUTseq libraries, which included unique molecular identifiers (UMIs), we deduplicated the reads using *UMI-tools* (v1.1.1).^19^ For libraries prepared for targeted gene sequencing, we deduplicated the reads using the *Agilent Genomics NextGen Toolkit* (AGeNT) (v2.0.5). For all the other libraries, we deduplicated the reads using the *MarkDuplicates* tool in the *Genome Analysis ToolKit* (GATK, v4.1.4.1).^20^ Finally, for libraries used for single-nucleotide variant (SNV) calling, we recalibrated the base scores using the *BQSRPipelineSpark* command in GATK, following the Broad Institute’s best practices.^21^

#### Copy Number Calling

To determine DNA copy number levels, we used the R package *QDNAseq*,^22^ which is optimized for FFPE samples, and *CNVkit*.^23^ Unless otherwise specified, we binned the genome in 50 kilobase (kb) windows. We plotted genome-wide copy number profiles by aggregating together the profiles of individual chromosomes using custom scripts in R available at https://github.com/ljwharbers/metastatic_lungcancer. We called amplifications (AMP) and deletions (DEL) using a threshold of the log_2_ ratio equal to 0.32 and −0.42, respectively. To determine the fraction of the genome with AMP or DEL, we calculated the percentage of 50 kb genomic windows that were called either as amplified or deleted using custom scripts in R available at https://github.com/ljwharbers/metastatic_lungcancer. To determine alterations in cancer-related genes, we overlapped the regions classified as amplified or deleted with the COSMICplus gene list available in the Supplementary Materials. To determine significant focal amplification and deletion events, we used *GISTIC2*^24^ with default settings.

#### SNV and Indel Calling

We analyzed WES and targeted sequencing data using *GATK* (v4.1.4.1) following the Broad Institute’s best practices. In the case of WES data, we used *GATK Mutect2* with primary tumor samples as a reference to call BM-specific SNVs and small insertions and deletions (indels). We then removed false-positive calls owing to sequence artifacts and contamination with the following GATK commands (in order): *LearnReadOrientationModel*; *GetPileupSummaries*; *CalculateContamination*; and *FilterMutectCalls*. Last, we annotated the filtered calls using the *GATK* command *Funcotator*. To identify potential pathogenic alterations, we used CHASMplus with two NSCLC annotators (*chasmplus_GBM* and *chasmplus_LGG*) and two brain tumor annotators (*chasmplus_LUAD* and *chasmplus_LUSC*).

#### Code Availability

All the custom codes used to process and analyze the sequencing data are available at the following GitHub link: https://github.com/ljwharbers/metastatic_lungcancer.

#### Data Availability

In line with the privacy regulations of the European Union and Sweden, the raw sequencing data generated in this study cannot be made publicly available. A detailed summary of sequencing statistics for each data set is available in the Supplementary Materials.

## Results

### NSCLC-BM Harbor a Higher Burden of SCNAs Compared to Matched Primary Tumors

**Figure 1:**
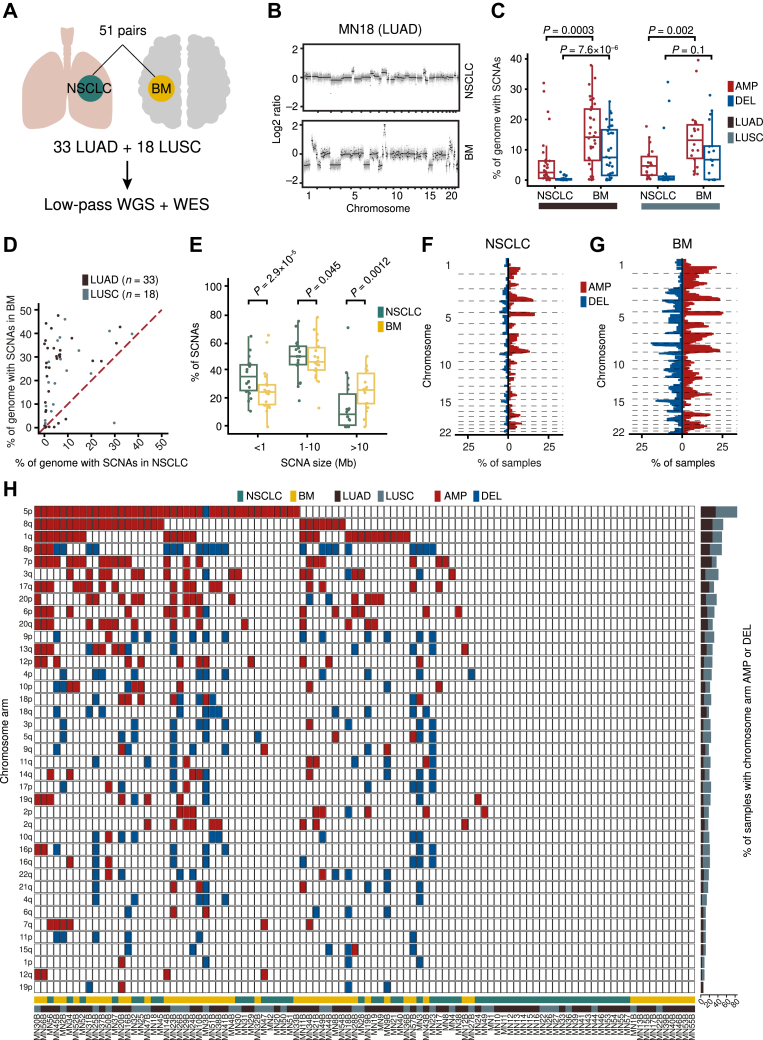
NSCLC-BM harbor significantly more SCNAs compared to the primary tumor samples. (*A*) Schematic representation of the discovery cohort and of the genomic assays performed on it. (*B*) Example of genome-wide SCNA profiles (50 kb resolution) for one LUAD-BM pair from one patient (MN18) in the discovery cohort. Each gray dot represents a 50 kb genomic bin. Black dots mark copy number levels determined by circular binary segmentation.^66^ (*C*) Percentage of the genome amplified or deleted in each primary tumor and BM sample in the discovery cohort, separately for LUAD and LUSC. Each dot represents one sample. *p*, Wilcoxon test, two-tailed. (*D*) Correlation of the percentage of the genome either amplified or deleted between the 51 (*n*) pairs of primary NSCLC and BM samples in the discovery cohort. Each dot represents one sample pair. The dashed red line represents the bisector of the angle between the x- and y-axis. (*E*) Distributions of the length of genomic segments amplified or deleted in each primary tumor and BM sample in the discovery cohort, separately for focal (<1 Mb), medium (1–10 Mb), and large (>10 Mb) SCNAs. *p*, Wilcoxon test, two-tailed. (*F*) Percentage of primary tumor samples (*n*) in the discovery cohort having consecutive 50 kb genomic bins (vertical axis) amplified or deleted. (*G*) Same as in (*F*) but for the corresponding BM samples. In all boxplots in (*C*) and (*E*), each box ranges from the 25th to the 75th percentile, the horizontal line marks the median value, and the whiskers span from the minimum to the maximum value. AMP, amplified; BM, brain metastases; DEL, deleted; kb, kilobase; LUAD, lung adenocarcinoma; LUSC, lung squamous cell carcinoma; Mb, megabase; SCNA, somatic copy number alteration; WES, whole-exome sequencing; WGS, whole-genome sequencing.

To identify novel pathogenic genomic alterations in NSCLC-BM, we retrospectively collected 51 pairs of FFPE primary NSCLC and BM samples from 33 patients with LUAD and 18 patients with LUSC (hereafter named “discovery cohort”) (Fig. 1*A* and Supplementary Table 1). We first profiled SCNAs in these samples by performing low-pass WGS and determining DNA copy number levels using two different callers (QDNAseq^22^ and CNVkit^23^) (Supplementary Table 2). Because both callers yielded similar copy number profiles (mean Pearson’s correlation coefficient [PCC]: 0.92 at 50 kb resolution), we decided to conduct all subsequent analyses using QDNAseq given that this caller is tailored for FFPE samples^22^ (Supplementary Fig. 1*A* and *B*). Visual inspection of the copy number profiles revealed that BM had consistently more SCNAs than the corresponding primary tumors (Fig. 1*B*). Accordingly, the SCNA burden (i.e., the fraction of the genome amplified or deleted) was significantly higher in BM, independently of the primary tumor histological diagnosis (Fig. 1*C* and *D*, Supplementary Fig. 1*C*–*F*, and Supplementary Table 3). Most SCNAs were medium sized (1–10 megabase [Mb]), followed by focal (<1 Mb) and large (>10 Mb) alterations (Fig. 1*E*). BM had a significantly higher number of large alterations compared with primary tumors, whereas the latter harbored significantly more focal SCNAs (Fig. 1*E*). Amplifications were more frequent along the q-arm of chromosome (chr) 1, 3, 8, and 17 and along the p-arm of chr5, 7, and 20, whereas deletions were more frequent on chr3p, 4p, 5q, 8p, 9p, and 18q (Fig. 1*F* and *G*). Recurrent BM-associated amplifications on chr5p and 8q were also previously identified in a small cohort of seven primary lung cancer and BM pairs profiled by WES.^25^

To corroborate these results, we applied an orthogonal nonsequencing-based method (NanoString^26^) to quantify the copy number of 87 genes frequently amplified or deleted in human cancers, in four NSCLC-BM pairs and one additional BM sample in our discovery cohort, for which we had enough gDNA left (Supplementary Table 4). We found a strong correlation (PCC: 0.62) between the NanoString counts in these four NSCLC-BM pairs and the expected versus observed read count log_2_ ratio of the genomic bins encompassing the genes assayed by NanoString (Supplementary Fig. 1*G*). The NanoString counts were significantly higher (*p* < 2.2 × 10^−16^, Wilcoxon test, two-tailed) for the amplified genes compared with the genes with a neutral copy number. In turn, the latter genes had significantly higher (*p* = 1.1 × 10^−7^, Wilcoxon test, two-tailed) NanoString counts compared with the deleted genes (Supplementary Fig. 1*H*). Together, these results indicate that NSCLC-BM tend to harbor substantially more SCNAs compared with their matched primary tumors.

### SCNAs Are Spatially Homogeneous in NSCLC-BM

**Figure 2:**
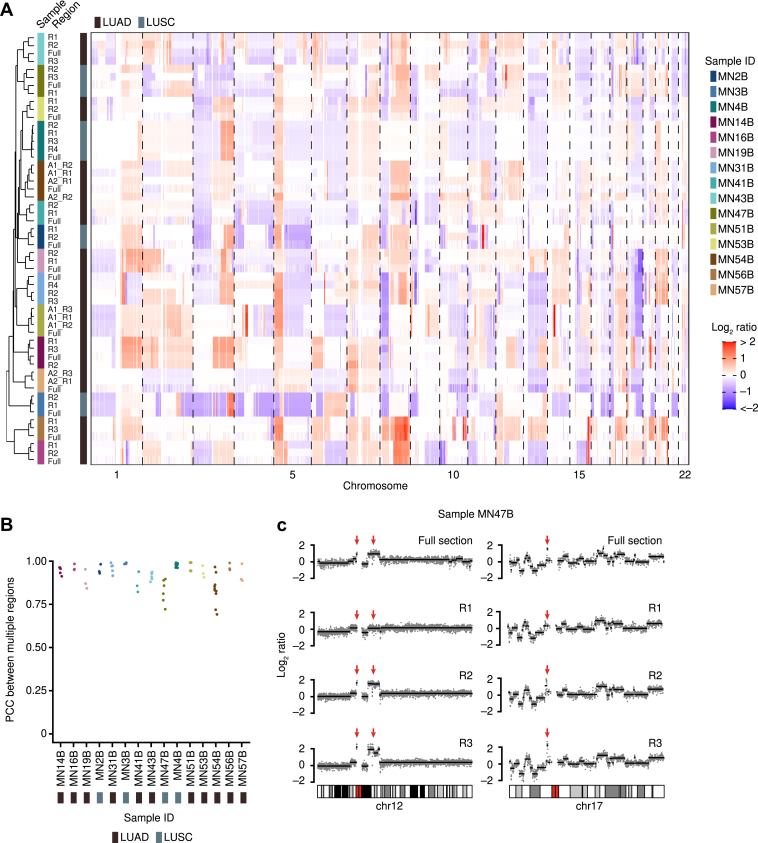
SCNAs are spatially homogeneous across NSCLC-BM. (*A*) Clustered heatmap of the observed versus expected read count log_2_ ratio in consecutive 50 kb genomic bins for multiple tissue regions in 15 BM samples from the discovery cohort (see Supplementary Fig. 2 for a map of the regions profiled). Individual regions profiled in each sample are labeled as “_R,” “A1,” and “A2” which refer to spatially distinct metastatic lesions. Sample IDs are the same as in Supplementary Table 1. (*B*) PCC of the genome-wide copy number levels between all possible pairs of tissue regions profiled in each BM sample found in (*A*). (*C*) Example of copy number profiles along chr12 and 17 in three tissue regions profiled by CUTseq^16^ inside a single FFPE tissue section from BM sample MN47B (see Supplementary Table 1). The profiles on the top row correspond to a full FFPE tissue section adjacent to the one in which gDNA was extracted from the regions displayed. Overall, the profiles are largely conserved between regions. The red arrows indicate clear differences between regions. BM, brain metastases; chr, chromosome; FFPE, formalin-fixed, paraffin-embedded; gDNA, genomic DNA; ID, identification; kb, kilobase; LUAD, lung adenocarcinoma; LUSC, lung squamous cell carcinoma; PCC, Pearson’s correlation coefficient; R, region; SCNA, somatic copy number alteration.

Primary tumors, including NSCLC, are typically characterized by high spatial heterogeneity of SCNAs and mutations, as revealed by multiregion sequencing performed in different tumor types.^27^, ^28^, ^29^, ^30^ In contrast, metastases seem to be less spatially heterogeneous than their corresponding primary tumors.^31^^,^^32^ The lower spatial heterogeneity of SCNAs or mutations in metastases is consistent with a scenario in which metastases form relatively late during tumor evolution, leaving a shorter time for genetic drift and spatial diversification to occur. To test how SCNAs are spatially distributed in NSCLC-BM, we profiled SCNAs in two to four spatially separated and relatively large (25–50 mm^2^) regions in FFPE tissue sections from 15 NSCLC-BM samples in the discovery cohort, leveraging the CUTseq method that we previously developed for multiregion SCNA profiling in FFPE tissue sections^16^ (Supplementary Fig. 2, Supplementary Table 2). The SCNA profiles of all the regions from the same tissue section clustered together with the corresponding SCNA profile obtained from gDNA extracted from an adjacent tissue section (Fig. 2*A*). Accordingly, the pairwise correlations between the SCNA profiles of all the regions in the same tissue section were typically very high (PCC > 0.90), except for three BM samples (MN41B, MN47B, and MN54B) for which some of the pairwise correlations dropped (Fig. 2*B*). Visual inspection of the SCNA profiles in these samples revealed that, indeed, some SCNA events were private to some regions and were not detected in the gDNA extracted from other regions in the same section or from an adjacent tissue section (Fig. 2*C*). These findings, together with the observation that NSCLC-BM harbor significantly more SCNAs compared with their matched primary tumors, suggest that SCNAs might form relatively late during the evolution of NSCLC. In contrast, in other tumor types, such as breast and prostate cancers, SCNAs have been proposed to form in bursts in the early stages of tumor evolution and are conserved between primary tumors and metastases.^33^^,^^34^

### Identification of Putative NSCLC-BM Driving SCNAs

**Figure 3:**
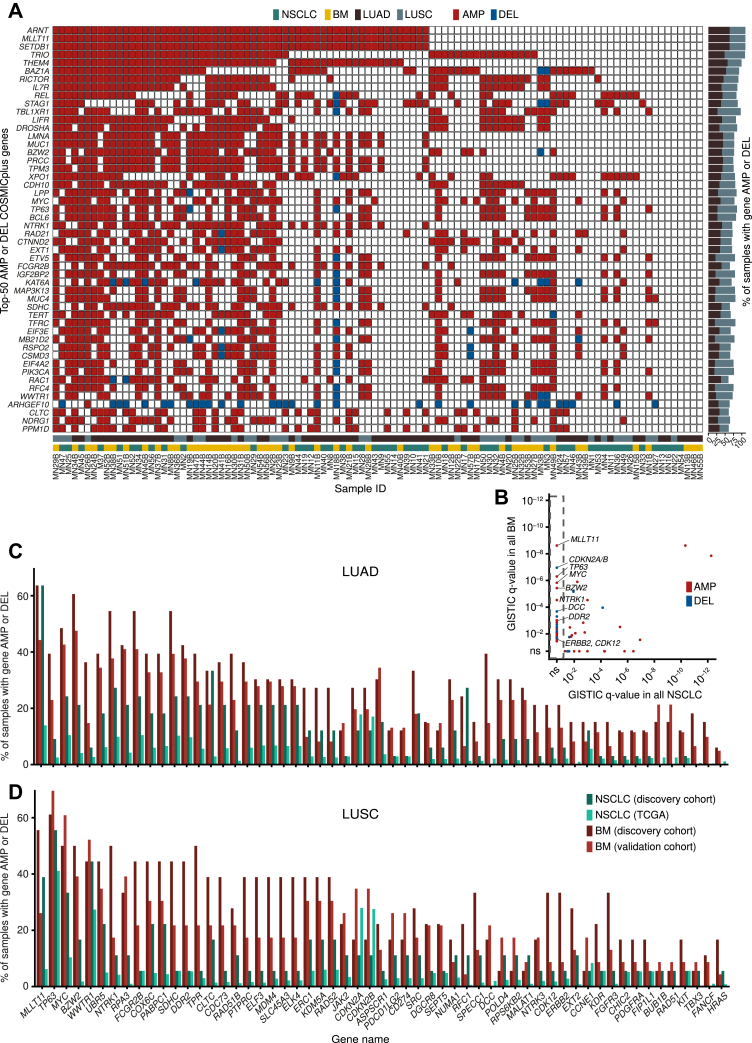
Comparison of SCNA profiles between matched primary tumor and BM samples allows identifying putative NSCLC-BM–driving genes. (*A*) Copy number status of the 50 most frequently amplified or deleted genes in the COSMICplus list (see Supplementary Table 3) across all the samples included in the discovery cohort. Sample IDs are the same as in Supplementary Table 1, and primary tumors (yellow) and BM (green) from each pair are illustrated adjacently. (*B*) q value assigned by GISTIC^24^ to each gene in the COSMICplus list (see Supplementary Table 3) in each pair of primary tumor and BM samples in the discovery cohort. The genes inside the dashed rectangle on the left have a not significant (ns) q value in the primary tumors and a significant q value in the BM samples, indicating they are significantly more amplified or deleted in BM than in the matched primary tumors. Only COSMICplus genes that have a significant q value in at least one sample type are found. The three most frequently amplified and deleted genes in BM and actionable genes significantly amplified in BM are indicated. (*C*, *D*) Percentage of samples in which the indicated genes are amplified or deleted in LUAD (*C*) and LUSC (*D*) samples from the discovery cohort and the validation cohort (84 BM samples) profiled by CUTseq^16^ (see Methods) or TCGA. AMP, amplified; BM, brain metastases; DEL, deleted; kb, kilobase; ID, identification; LUAD, lung adenocarcinoma; LUSC, lung squamous cell carcinoma; SCNA, somatic copy number alteration.

We then sought to identify SCNAs that might drive the formation of NSCLC-BM by amplifying or deleting known cancer genes. We first manually curated a list of cancer-associated genes comprising all 720 genes listed in the Catalogue of Somatic Mutations in Cancer (COSMIC)^35^ and 58 additional genes that have been previously associated with NSCLC (Supplementary Table 5). We hereafter refer to this gene list as “COSMICplus.” Among these genes, the five most frequently amplified genes in the discovery cohort were *ARNT* (63.6% of LUAD and 47.2% of LUSC samples), *MLLT11* (63.6% and 47.2%), *SETDB1* (63.6% and 47.2%), *TRIO* (61.1% and 48.5%), and *THEM4* (57.6% and 41.7%), whereas *ARHGEF10* (30.3% and 30.5%), *ISX* (24.2% and 19.4%) *CDKN2A/B* (19.7% and 13.9%), *DCC* (19.7% and 8.3%), and *PHLPP1* (12.1% and 5.6%) were the most frequently deleted genes (Fig. 3*A*).

To identify plausible SCNAs underlying BM formation in NSCLC, we reasoned that cancer genes that are more frequently amplified or deleted in BM in comparison to the corresponding primary tumors could be considered as candidate pathogenic alterations in NSCLC-BM. To identify such genes, we applied Genomic Identification of Significant Targets in Cancer (GISTIC)^24^ to identify genomic regions significantly focally amplified or deleted across the 51 primary tumor and BM samples in the discovery cohort. Intersection of the identified regions with the COSMICplus cancer gene list (Supplementary Table 5) revealed 45 and 12 genes that were significantly focally amplified and deleted, respectively, in BM but not in the corresponding primary tumors (Fig. 3*B* and Supplementary Table 6). The same genes were altered infrequently (comparable with the alteration frequency in primary tumors in the discovery cohort) in 1017 primary NSCLC tumors sequenced in The Cancer Genome Atlas (TCGA), including 516 LUAD and 501 LUSC samples (Fig. 3*C* and *D*), further indicating that their alteration is specific to BM. The three most significantly amplified genes in BM were *MLLT11* (60.8% of BM versus 54.9% of NSCLC), *TP63* (47.1% versus 25.5%), and *MYC* (49% versus 27.5%), whereas the three most significantly deleted genes in BM were *CDNK2A/B* (23.5% versus 11.8%), *DCC* (31.4% versus 0%), and *TBX3* (13.8% versus 0%). Importantly, *MYC* amplifications and *CDKN2A/B* deletions were previously identified as putative BM drivers by applying GISTIC to a cohort of 73 matched BM and primary LUAD samples,^10^ highlighting the validity of our approach. Notably, among genes significantly more frequently amplified in BM, we found several genes that could be directly targeted with available drugs, including *CDK12* (amplified in 21.2% of LUAD-BM and 33.3% of LUSC-BM versus 6.1% and 5.6% of LUAD and LUSC primary tumors, respectively), *DDR2* (42.4% and 44.4% versus 24.2% and 5.6%), *ERBB2* (21.2% and 33.3% versus 6.1% and 5.6%), and *NTRK1* (54.6% and 50% versus 27.3% and 11.1%).

To corroborate these findings, we profiled SCNAs in an independent cohort of 84 BM samples from 23 LUSC and 61 LUAD patients (hereafter named “validation cohort”), using the CUTseq method that we previously developed^16^ (Supplementary Tables 2 and 7). In agreement with our findings in the discovery cohort, amplifications were also significantly more frequent than deletions in the validation cohort, independently of the primary tumor-histological diagnosis (Supplementary Fig. 3*A* and *B*). Moreover, the percentage of samples carrying amplifications or deletions of COSMICplus genes was strongly correlated (PCC: 0.95) between the discovery and validation cohorts (Supplementary Fig. 3*C*). The correlation was even higher (PCC: 0.98) for putative BM-driving genes that were significantly more frequently amplified or deleted in BM compared with their matched primary tumors (Supplementary Fig. 3*D*). Altogether, these results indicate that SCNAs affecting specific cancer genes might represent pathogenic alterations that underlie the formation or progression of BM in patients with NSCLC and that the product of some of these genes might be targeted with available drugs.

### Identification of Putative NSCLC-BM Driving Mutations

**Figure 4:**
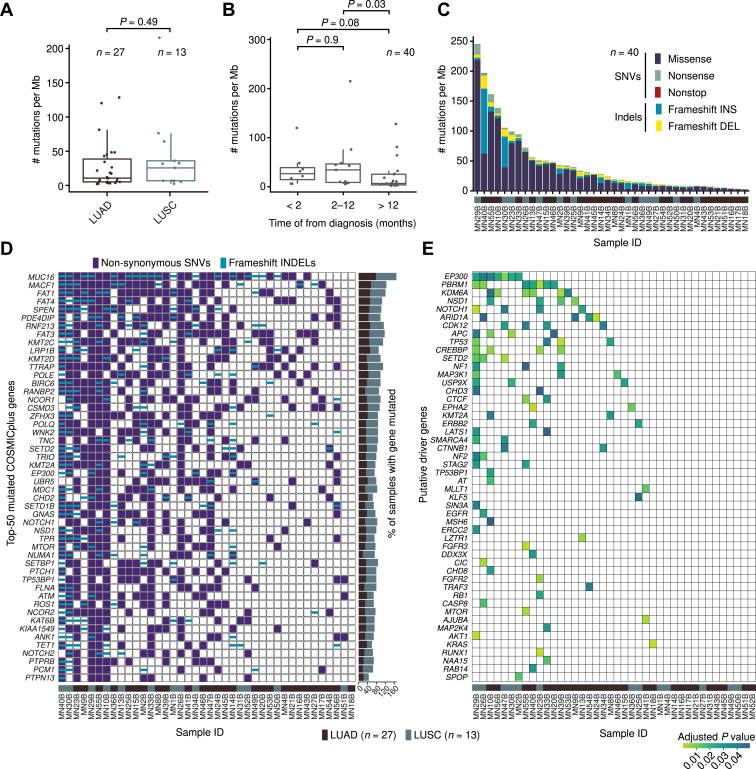
WES of matched primary tumor and BM samples allows identifying NSCLC-BM putative–driving mutations. (*A*) Tumor mutation burden in 40 BM samples in the discovery cohort profiled by WES. *p*, Wilcoxon test, two-tailed. (*B*) Same as in (*A*) but stratifying patients on the basis of the timing of BM diagnosis. (*C*) Number of different mutation types in each of the 40 BM samples profiled by WES. (*D*) Top-50 COSMICplus (see Supplementary Table 5) mutated genes in the 40 BM samples in the discovery cohort profiled by WES. (*E*) COSMICplus genes annotated as high-confidence, BM-specific pathogenic alterations on the basis of (CHASM).^42^ The sample IDs in (*C*) and (*D*) are the same as in Supplementary Table 1. BM, brain metastases; ID, identification; indels, small insertions and deletions; LUAD, lung adenocarcinoma; LUSC, lung squamous cell carcinoma; SNV, single-nucleotide variant; WES, whole-exome sequencing.

Last, we turned our attention to SNVs and small indels, aiming at identifying putative pathogenic alterations in NSCLC-BM in addition to SCNAs. To this end, we performed WES on 40 of 51 NSCLC-BM sample pairs in the discovery cohort for which there was enough gDNA left, including 27 LUAD and 13 LUSC samples (Supplementary Table 2). Because germline gDNA had not been collected from these patients, we used the primary tumor samples as a reference for calling SNVs and indels in the corresponding BM samples. The mutations identified through this approach can thus be considered as BM-specific events. We found that the metastasis mutation burden was comparable between LUAD and LUSC samples (Fig. 4*A*). Late-onset BM (i.e., metastases detected >12 mo after diagnosis of the primary tumor) had a significantly lower mutation burden than synchronous (<2 mo) or early onset (2–12 mo) BM (Fig. 4*B*), suggesting that early and late metastases might be driven by different mutational processes. Most of the mutations identified were missense substitutions (mainly C > T and C > A), and in a few cases (MN1B, MN14B, MN30B, MN40B), frameshift indels represented the dominant mutation type (Fig. 4*C* and Supplementary Fig. 4*A*). The most common mutational signatures^36^ were signatures 1, 3, 4, and 6 (Fig. 4*D* and Supplementary Fig. 4*B*). As expected, signature 4, which is associated with tobacco exposure, was overrepresented in patients with smoking history (Supplementary Fig. 4*C*).

We then assessed which genes, among those listed in the COSMICplus list, were most often mutated in the 40 NSCLC-BM samples sequenced by WES. Among the most frequently mutated genes, we identified several genes directly or indirectly implicated in cell motility. These included *MUC16* (77.8% of LUAD-BM and 92.3% of LUSC-BM), which encodes a large cell surface protein and is best known as a biomarker (CA-125) for ovarian cancer^37^; *MACF1* (55.6% and 69.2%), which encodes a large intracellular protein that bridges actin and microtubule filaments^38^; and *FAT1* (51.9% and 69.2%), *FAT3* (48.1% and 69.2%), and *FAT4* (51.9% and 53.8%), which encode three members of the cadherin family of transmembrane proteins previously implicated in the regulation of cell motility^39^ (Fig. 4*D*). Other frequently mutated genes included several genes encoding DNA or histone-modifying enzymes, such as *KMT2A* (37% of LUAD-BM and 38.5% of LUSC-BM), *KMT2C* (48.1% and 61.5%), *KMT2D* (37% and 61.5%), *SETD2* (37% and 46.2%), and *SETD1B* (29.6% and 38.5%), which encode various histone methyltransferases^40^; *EP300* (33.3% and 38.5%), which encodes a histone acetyltransferase playing a pivotal role in transcription regulation^41^; and *KAT6B* (33.3% and 38.5%), which also encodes a histone acetyltransferase (Fig. 4*D*). Notably, *KMT2C* mutations were previously detected in 50% of BM samples in a Chinese cohort of 12 matched NSCLC and BM profiled by WES,^11^ which is in line with the frequency measured in our cohort. Another gene frequently mutated in BM reported in the same study was *PDE4DIP* (25% of BM samples), which encodes a phosphodiesterase 4D interacting protein and was mutated in 44.4% of LUAD-BM and 53.8% of LUSC-BM in our cohort.

To identify high-confidence putative NSCLC-BM driving mutations, we then applied cancer-specific high-throughput annotation of somatic mutations (CHASM)^42^ to the list of the identified mutations. In 26 of 40 (65%) NSCLC-BM samples sequenced, we identified such mutations in at least one gene (Fig 4*E*). Among 50 high-confidence NSCLC-BM–specific pathogenic alterations identified by CHASM, many affected genes were involved in chromatin editing and remodeling and in three-dimensional (3D) genome organization. These included *EP300* (putative pathogenic alterations detected in two of 27 LUAD-BM [7.4%] and in eight of 13 LUSC-BM [61.5%] samples); *PBMR1* (14.8% and 23.1%), which encodes a component of the SWI/SNF chromatin remodeling complex^43^; *KDM6A* (25.9% and 0%); *CREBBP1* (7.4% and 23.1%), which encodes a master transcription coactivator frequently mutated in leukemias^44^; *KMT2A* (7.4% and 0%); *CTCF* (7.4% and 0%), which encodes a transcription factor crucial for structuring chromatin loops along the genome^45^; and *STAG2* (3.7% and 7.7%), which encodes a subunit of the cohesin complex, that is, also crucial for shaping chromatin loops and is mutated in many cancer types^46^ (Fig 4*E*). Accordingly, gene ontology analysis revealed significant enrichment of terms related to transcription and chromatin remodeling, but also of terms associated with cytoskeleton and cell motility (Supplementary Fig. 4*D*). Notably, two LUAD-BM (15.4%) and three LUSC-BM (11.1%) samples contained pathogenic alterations in *CDK12*, further implicating this potentially targetable gene in the pathogenesis of NSCLC-BM.

To corroborate these findings, we performed targeted sequencing of 115 additional BM samples including 61 LUAD-BM and 23 LUSC-BM (validation cohort) using the Glasgow Cancer Core Panel covering 174 genes frequently mutated in solid tumors (Supplementary Tables 7 and 8). Among the frequently mutated genes, we again found multiple genes encoding chromatin-modifying enzymes, including *KMT2A* (30.1% of LUAD-BM and 28.1% of LUSC-BM), *EP300* (25.3% and 21.9%), *SETD2* (20.5% and 9.4%), *CREBBP1* (18.1% and 21.9%), *STAG1* (9.6% and 15.6%), and *STAG2* (12% and 15.6%) (Supplementary Fig. 5*A*). Importantly, we observed a strong correlation (PCC: 0.82) between the frequency of mutation in genes covered by the panel in the discovery and validation cohorts (Supplementary Fig. 5*B*). The correlation was even stronger (PCC: 0.89) for the subset of high-confidence putative NSCLC-BM–driving alterations identified by CHASM, which were also covered by the gene panel (Supplementary Fig. 5*C*). Altogether, these results suggest that mutations in multiple genes involved in chromatin editing and genome architecture might play a role in the pathogenesis of BM in a subset of patients with NSCLC. Future studies are needed to corroborate these findings in larger cohorts and to investigate the impact of these mutations on chromatin architecture and gene expression regulation in NSCLC cells.

## Discussion

Our study further expands the existing compendium of genomic alterations underlying NSCLC-BM, highlighting the importance of comparative genomic profiling of matched primary and metastatic tumors to identify putative pathogenic alterations underlying metastasis. The list of presumably pathogenic alterations that we have identified in NSCLC-BM represents a powerful resource for designing future studies exploring the molecular pathogenesis and targetability of NSCLC-BM.

Although BM remain a major cause of morbidity and mortality in patients with NSCLC, the genomic landscape of NSCLC-BM has not been thoroughly characterized. A previous study conducted on 73 pairs of primary LUAD and matched BM samples identified amplifications of *MYC*, *YAP1*, and *MMP13* and deletions of *CDKN2A* as putative LUAD-BM–specific pathogenic alterations. Moreover, it has been found that simple overexpression of three putative LUAD-BM drivers (*MYC*, *YAP1*, and *MMP13*) in patient-derived mouse xenografts increases the incidence of BM.^10^ Nevertheless, that study did not include LUSC-BM and did not identify any potentially targetable BM-specific pathogenic alterations. In another genomic study of BM of various origin, several potentially actionable genomic alterations were associated with lung cancer BM, including *AKT1*, *AURKA*, *CDK6*, *EGFR*, *MEK1*, *MET*, and *PIK3CA* gene amplifications and *CDKN2A* deletions.^8^ Nevertheless, this study did not compare matched primary NSCLC and BM samples. Here, we have identified several plausible pathogenic alterations underlying LUAD-BM and LUSC-BM that affect potentially targetable genes, including *CDK12*, *DDR2*, *ERBB2*, and *NTRK1*. NSCLC-initiating driver events, such as alterations affecting *EGFR* and *ALK* genes, are highly concordant between primary NSCLC and matched BM lesions.^47^ The initiating drivers could be early (clonal) genomic events during the establishment of primary NSCLC, and simultaneously they might be involved in NSCLC dissemination to the brain.^48^ Nevertheless, in NSCLC tumors carrying wild-type *EGFR* and *ALK* genes, the brain-seeding metastatic cells acquire additional genetic alterations during the early stages of tumor evolution.^48^ This subclonal divergence seems to be crucial in the spreading of NSCLC tumor cells to the brain. Thus, identifying pathogenic alterations underlying NSCLC-BM is essential toward preventing or delaying the spread of cancer cells to the brain.

Multiple clinical trials are currently evaluating the efficacy of various drugs targeting the products of *CDK12*, *DDR2*, *ERBB2*, and *NTRK1*, including dinaciclib (CDK12); dasatinib (DDR2); lapatinib, afatinib, dacomitinib, neratinib, or pyrotinib (ERBB2); and entrectinib, larotrectinib, or repotrectinib (NTRK1). Thus, new trials could be readily designed to assess the efficacy of these agents in patients with NSCLC with BM harboring *CDK12*, *DDR2*, *ERBB2*, or *NTRK1* amplifications or mutations. DDR2, ERBB2, and NTRK1 inhibitors have been proven effective against primary tumors harboring *DDR2* or *ERBB2* mutations or *NTRK1* rearrangements, including NSCLC tumors.^49^ Nevertheless, the activity of these agents against NSCLC-BM with amplifications of these genes has not been assessed. Of note, *ALK*, *NTRK*, *RET*, and *ROS1* targeting agents were found to have promising activity in the treatment of NSCLC-BM^50^, ^51^, ^52^ and might be considered as first-line therapy in lieu of radiotherapy or neurosurgery in selected patients.^7^ Hence, expanding the repertoire of targetable NSCLC-BM genomic alterations—as we did in this study—can pave the way to follow-up clinical studies aiming at establishing novel therapeutic opportunities for patients with NSCLC with intracranial disease.

From a clinical standpoint, obtaining BM tissue from patients with NSCLC is extremely difficult, and the main indications for surgery of BM are large lesions (>3 cm) with a mass effect, localization in the posterior fossa of the skull, risk of hydrocephalus, and cystic lesions with necrosis.^53^^,^^54^ Hence, NCSCL-BM samples such as those we have analyzed in this study are highly valuable. Importantly, several studies have found concordance of actionable mutations between primary NSCLC and BM lesions,^55^, ^56^, ^57^, ^58^ although other studies have reported discordant results.^59^^,^^60^ Therefore, when synchronous BM samples are not available, the choice of molecularly targeted therapy may be reasonably based on the results of genomic profiling of the primary tumor. Nevertheless, in the case of NCLSC-BM acquired during the treatment of the primary tumor, genomic profiling might reveal potentially targetable alterations that were not detected in the primary lesion (either because they were truly absent or because they were only present in a small tumor subclone). Therefore, whenever possible, asynchronous NSCLC-BM should be genomically profiled to maximize the chances of finding targetable alterations.

The implications of our results for immunotherapy or chemoimmunotherapy in patients with NSCLC with BM are less clear. The effectiveness of immunotherapy for patients with NSCLC with BM seems lower than that for other patients with NSCLC, probably owing to a more immunosuppressive environment in BM.^61^ Nevertheless, several clinical trials have revealed that BM status does not significantly influence the efficacy of immunotherapy in lung cancer, suggesting both BM and non-BM patients could obtain comparable benefits.^62^ Hence, further studies on the influence of the BM molecular background on the efficacy of immunotherapy in patients with NSCLC are necessary.

In addition to putative pathogenic alterations in NSCLC-BM that could be targeted by existing drugs, our study also revealed a substantial enrichment of SCNAs and mutations in genes encoding for histone methyltransferases (*KMT2A*, *KMT2C*, *KMT2D*, *SETD2*, *SETD1B*), histone acetyltransferases (*EP300*, *KAT6B*), chromatin remodeling factors (*PBMR1*), and 3D genome shapers (*CTCF*, *STAG1/2*) in NSCLC-BM compared with primary NSCLC tumors. Alterations in these genes might contribute to activating prometastatic gene expression programs in NSCLC cells by rewiring 3D genome domains, such as chromatin loops^63^ and topologically associating domains (TADs).^64^ Nevertheless, further studies are needed to investigate if and how alterations in these genes rewire the 3D genome landscape in NSCLC cells, aiming at uncovering potential vulnerabilities and identifying novel therapeutic targets.

Our study also contributes to shed light on the evolutionary history of BM in patients with NSCLC. By comparing SCNA profiles in matched NSCLC and BM samples, we found that the latter harbor a significantly higher burden of SCNAs, with amplifications dominating the genomic landscape of NSCLC-BM. This observation should be further validated on a wider array of samples from different clinical settings to uncover genetically favorable markers that might be potentially harnessed to prevent or delay the onset of BM. Furthermore, by profiling SCNAs across multiple regions in individual BM lesions, we found that the SCNA profiles measured at different locations within the same metastasis are largely similar. These observations are consistent with the parallel evolution model that was recently proposed as the preferred dissemination pattern for LUAD-BM^12^ and with the previous finding that spatially and temporally separated BM are genetically homogenous.^65^ Together, our observations are compatible with a tumor evolution model in which SCNAs form in a single burst in one or few tumor cells within the primary tumor mass, which then metastasize to the brain where they expand without further SCNA diversification, explaining the absence of SCNA spatial heterogeneity within BM. Alternatively, the latter could be explained by a rapid outgrowth of a single metastatic clone, leaving little time for new SCNAs to emerge in the population. One limitation of our multiregion SCNA profiling approach is that we could extract gDNA only from spatially distinct regions within a single thin tissue section. Therefore, we cannot exclude that by profiling additional tissue sections we would discover more heterogeneous SCNA patterns. Another limitation of our study is that all the samples were obtained from from a homogenous ethnic group (European Poles). Hence, larger multicentric studies including more diverse populations are needed to uncover the full spectrum of NSCLC-BM genomic alterations and potential therapeutic targets.

## CRediT Authorship Contribution Statement

**Marcin Nicoś:** Conceptualization, Methodology, Investigation, Data curation, Validation, Funding acquisition, Project administration, and Writing.

**Luuk Harbers:** Data curation, Formal analysis, Software, Visualization, and Writing.

**Enrico Patrucco, Maximilian Kramer-Drauberg, Claudia Voena:** In vitro and in vivo models (following the Reviewers’ recommendations, this part was removed from the final manuscript) and Writing.

**Xiaolu Zhang, Michele Simonetti:** Investigation and Writing.

**Anna Kowalczyk, Aleksandra Bożyk:** Clinical samples and Writing.

**Rafał Pęksa, Bożena Jarosz, Justyna Szumiło:** Pathologic annotation and Writing.

**Monika Żuk, Bartosz Wasąg:** Validation and Writing.

**Katarzyna Reszka:** Writing.

**Renata Duchnowska, Janusz Milanowski, Paweł Krawczyk:** Conceptualization and Writing.

**Roberto Chiarle:** In vitro and in vivo models (following the Reviewers’ recommendations, this part was removed from the final manuscript), Funding acquisition, and Writing.

**Magda Bienko:** Funding acquisition and Writing.

**Jacek Jassem:** Conceptualization, Funding acquisition, and Writing.

**Chiara Ambrogio:** In vitro and in vivo models (following the Reviewers’ recommendations, this part was removed from the final manuscript), Funding acquisition, Supervision, and Writing.

**Nicola Crosetto:** Conceptualization, Project administration, Visualization, and Writing.
