# Supplementary material for: Genomic Profiling Identifies Putative Pathogenic Alterations in NSCLC Brain Metastases
Source: JTO Clin Res Rep. 2022 Nov 11;3(12):100435. doi: 10.1016/j.jtocrr.2022.100435 (PMC9763853; doi:10.1016/j.jtocrr.2022.100435)
Supplement: Supplementary Material [file mmc1.docx]

**SUPPLEMENTARY INFORMATION**

**Genomic profiling identifies putative pathogenic alterations in non-small cell lung cancer brain metastases**

**Marcin Nicoś, Luuk Harbers, Enrico Patrucco, Maximilian Kramer-Drauberg, Xiaolu Zhang, Claudia Voena, Anna Kowalczyk, Aleksandra Bożyk, Rafał Pęksa, Bożena Jarosz, Justyna Szumiło, Michele Simonetti, Monika Żuk, Bartosz Wasąg, Katarzyna Reszka, Renata Duchnowska, Janusz Milanowski, Roberto Chiarle, Magda Bienko, Paweł Krawczyk, Jacek Jassem, Chiara Ambrogio & Nicola Crosetto**

1. Supplementary Figures pg. 2

2. Supplementary Tables pg. 8

**1. Supplementary Figures**

**
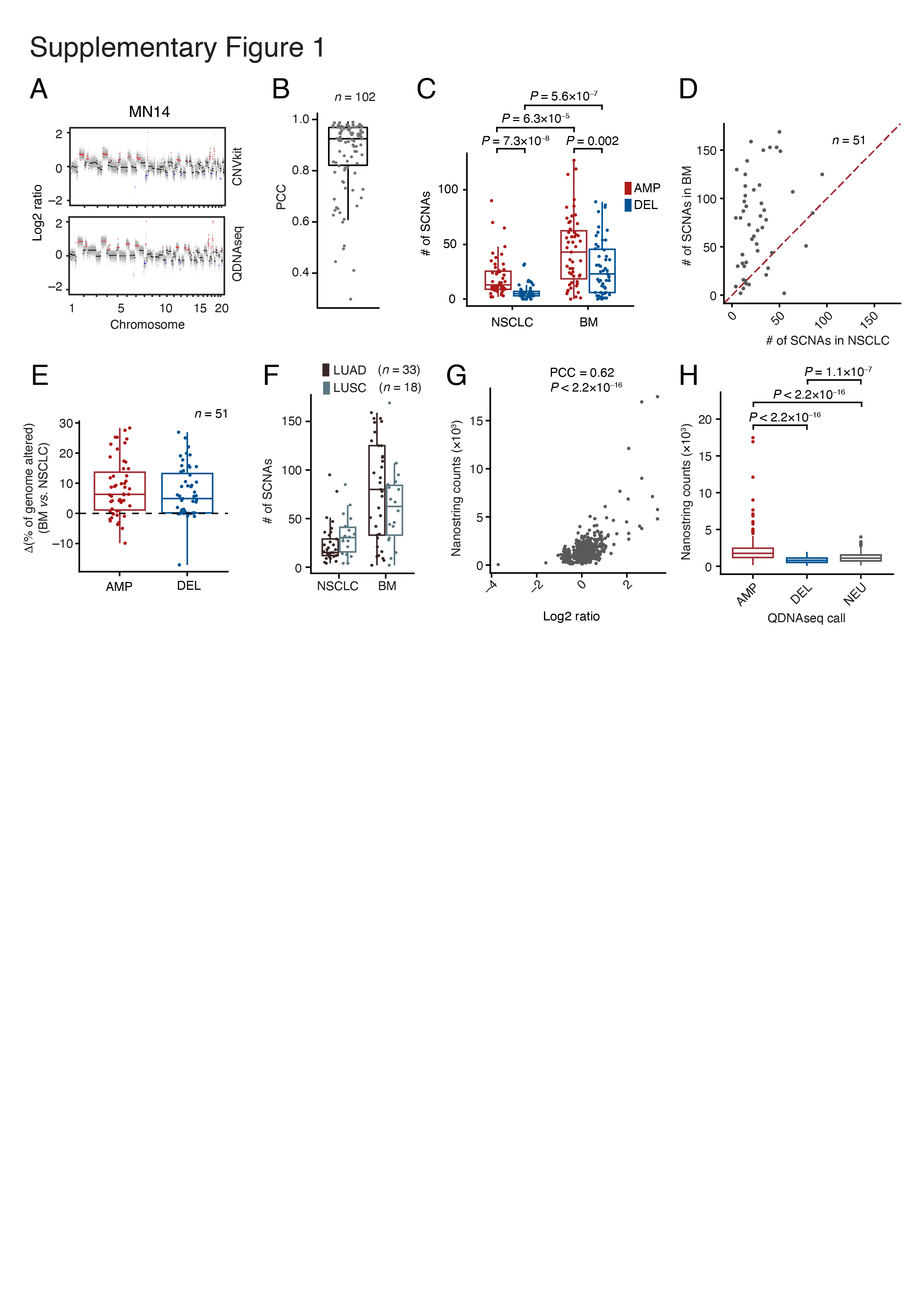
**

**Supplementary Fig. 1.** Profiling of SCNAs in matched NSCLC and BM samples. (**A**) Example of genome wide SCNA profiles (50 kilobase, kb resolution) determined using two different CNV callers (CNVkit and QDNAseq) for one NSCLC sample (MN14) in the discovery cohort (see **Supplementary Table 1**). Each grey dot represents a 50 kb genomic bin. Red dots, genomic bins with copy number gain. Blue dots, genomic bins with copy number loss. Black dots, genomic bins with neutral copy number. (**B**) Distribution of Pearson’s correlation coefficient (PCC) values between the copy number levels determined with CNVkit and QDNAseq for all the primary NSCLC and BM samples (*n*) in the discovery cohort. Each grey dot represents one NSCLC sample. (**C**) Distributions of the number of 50 kb genomic bins amplified (AMP) or deleted (DEL) in each primary tumor and BM sample in the discovery cohort. Each dot represents one sample. *P*, Wilcoxon test, two-tailed. (**D**) Correlation between the number of genomic segments either amplified or deleted in each of the 51 (*n*) pairs of NSCLC and BM samples in the discovery cohort. Each dot represents one sample. The dashed red line represents the bisector of the angle between the x- and y-axis. (**E**) Distributions of the differential (Δ) of the percentage of the genome amplified (AMP) or deleted (DEL) between a BM and the corresponding primary NCSCL sample, for each of the 51 (*n*) pairs of NSCLC and BM samples in the discovery cohort. Each dot represents one sample. (**F**) Distributions of the number of amplification and deletion events (SCNAs) in the 51 (*n*) pairs of NSCLC and BM samples in the discovery cohort, separately for lung adenocarcinomas (LUAD) and squamous cellular carcinomas (LUSC). Each dot represents one sample. (**G**) Correlation between the copy number determined by Nanostring and the expected vs. observed sequence read count log2 ratio of the corresponding genomic bin, for 87 cancer-associated genes assayed in 4 NSCLC-BM pairs and one additional BM sample in the discovery cohort. PCC, Pearson’s correlation coefficient. *P*, Wilcoxon test, two-tailed. Each dot represents one 50 kb genomic bin encompassing one of the 87 genes. (**H**) Distributions of the Nanostring counts for genomic bins classified as amplified (AMP), deleted (DEL), or copy number neutral (NEU) in the 4 NSCLC-BM pairs and one additional BM sample validated with Nanostring. *P*, Wilcoxon test, two-tailed. In all the boxplots in the figure, each box ranges from the 25^th^ to the 75^th^ percentile, the horizontal line marks the median value, and the whiskers span from the minimum to the maximum value.

**
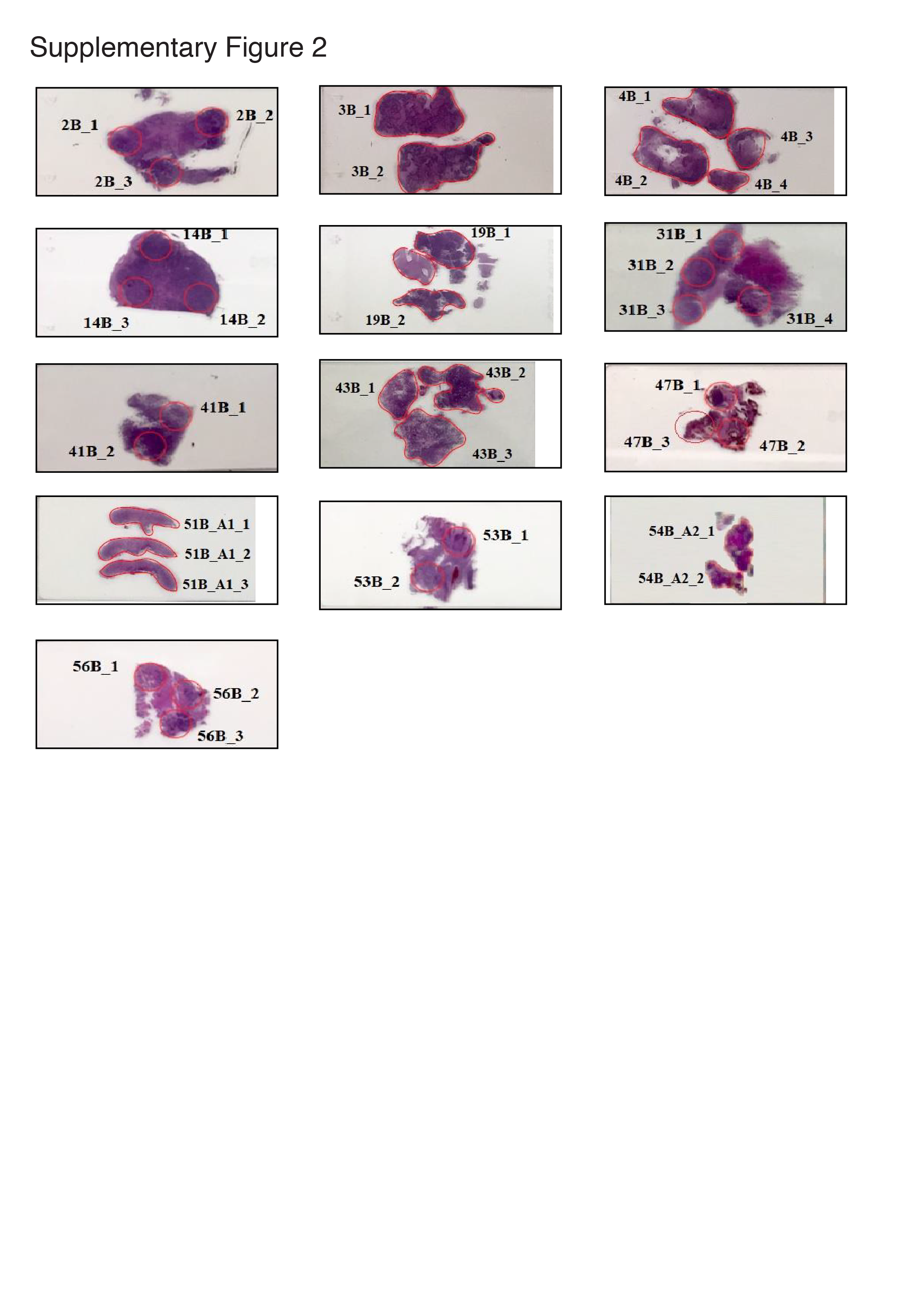
**

**Supplementary Fig. 2.** Multi-region SCNA profiling in BM. Hematoxylin-eosin-stained tissue sections used for multi-region SCNA profiling by CUTseq. The red lines mark the regions from which genomic DNA was extracted for CUTseq. Sample IDs are the same as in **Supplementary Table 1** (except the ‘MN’ in front of the number followed by ‘B’). The numbers refer to the number of regions profiled in each BM sample. Images are missing for two samples (MN16B and MN57B).


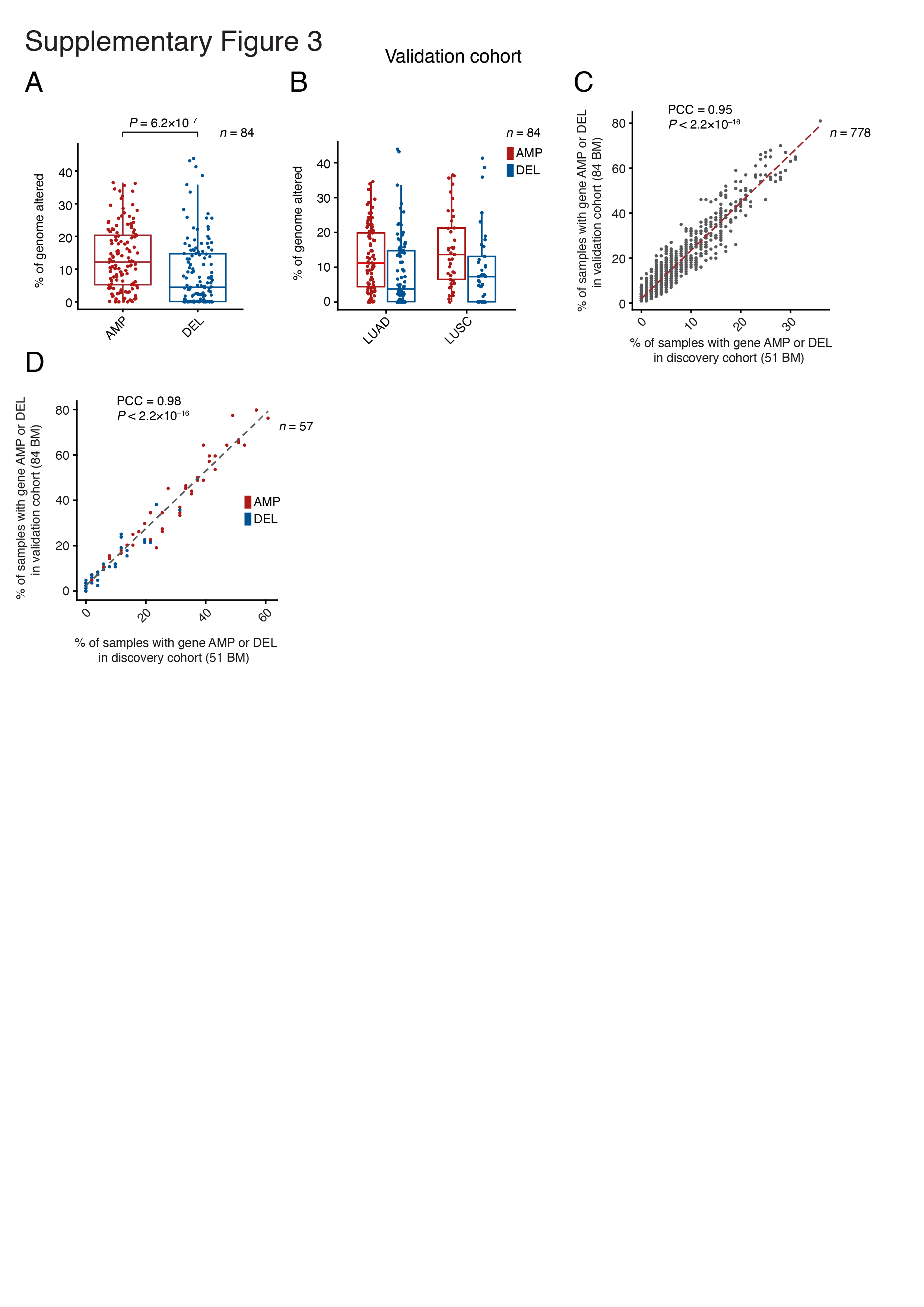


**Supplementary Fig. 3.** Validation of SCNAs in an independent cohort of BM samples. (**A**) Distributions of the percentage of the genome amplified (AMP) or deleted (DEL) in 84 (*n*) BM samples in the validation cohort profiled with CUTseq (see **Supplementary Table 7**). Each dot represents one sample. *P*, Wilcoxon test, two-tailed. In each boxplot, the box ranges from the 25^th^ to the 75^th^ percentile, the horizontal line marks the median value, and the whiskers span from the minimum to the maximum value. (**B**) Same as in (A) but separately for lung adenocarcinomas (LUAD) and squamous cellular carcinomas (LUSC). (**C**) Correlation between the percentage of samples that have one of the genes in the COSMICplus list (see **Supplementary Table 5**) either amplified or deleted in the discovery vs. validation cohort. PCC, Pearson’s correlation coefficient. *P*, Wilcoxon test, two-tailed. Each dot represents one of the 778 genes (*n*) in the COSMICplus list. Dashed red line, linear regression fit. (**D**) Same as in (C) but for 57 putative BM driving genes identified using GISTIC (see **Methods**).

**
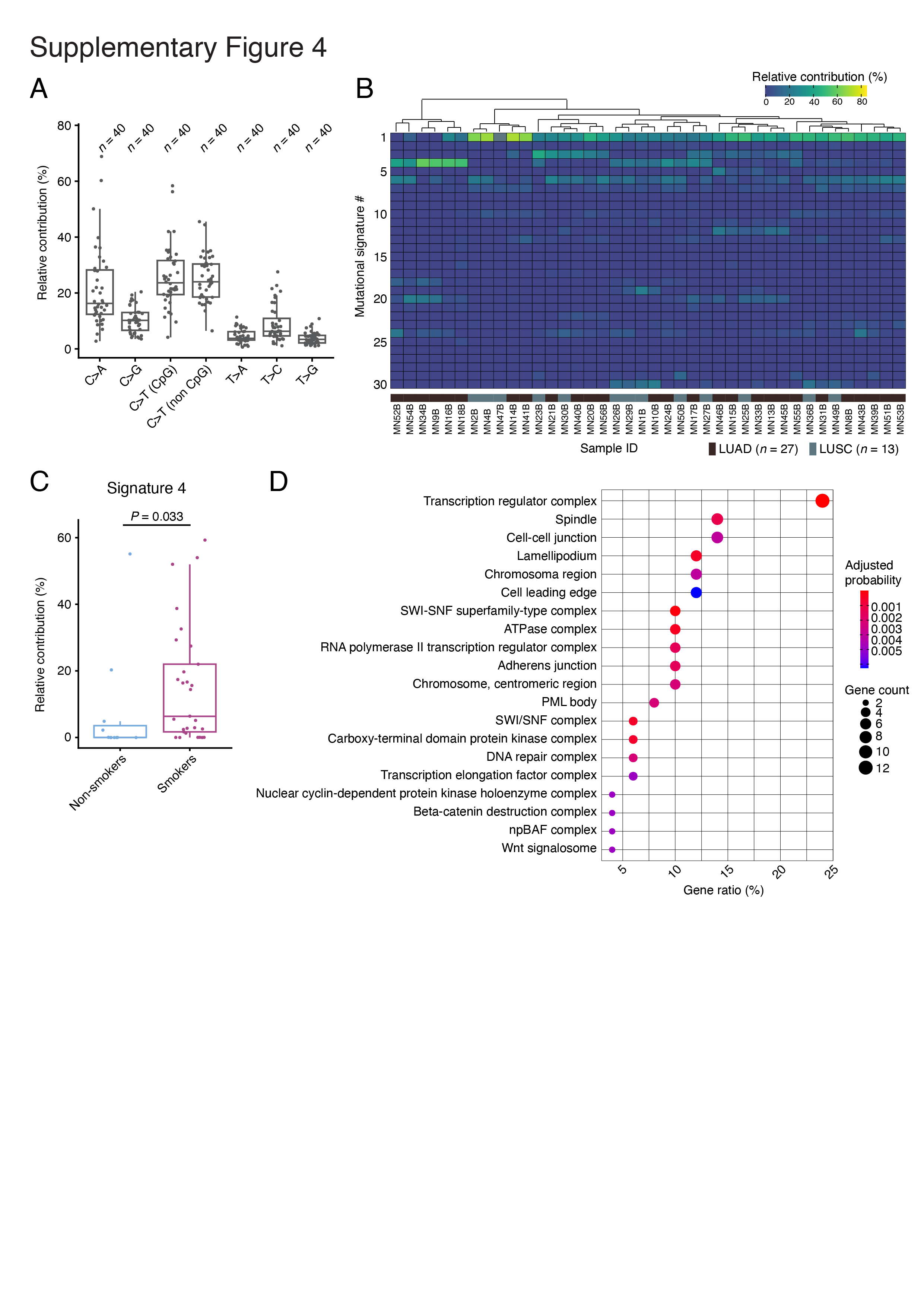
**

**Supplementary Fig. 4.** Mutational characteristics of 40 BM samples in the discovery cohort that were subjected to whole-exome sequencing. (**A**) Distributions of the relative frequency of different transitions and transversions detected in the 40 BM samples (*n*) using the paired NSCLC samples as a reference for calling single-nucleotide variants (SNVs) and indels. (**B**) Heatmap showing the relative importance of each of the 30 mutational signatures (rows) listed in the COSMIC Mutational Signatures database ([https://cancer.sanger.ac.uk/signatures/](https://cancer.sanger.ac.uk/signatures/#:~:text=COSMIC%20Mutational%20Signatures%20is%20a,wider%20Cancer%20Grand%20Challenges%20Project)), for each of the 40 BM samples in the discovery cohort that were profiled by WES (columns). Sample IDs are the same as in **Supplementary Table 1**. (**C**) Relative importance of signature #4 (tobacco signature) in BM samples from NSCLC patients with or without smoking history. *P*, Wilcoxon test, two-tailed. (**D**) Gene ontology analysis of putative BM driving pathogenic alterations identified using CHASM. Cellular compartment terms are shown.

**
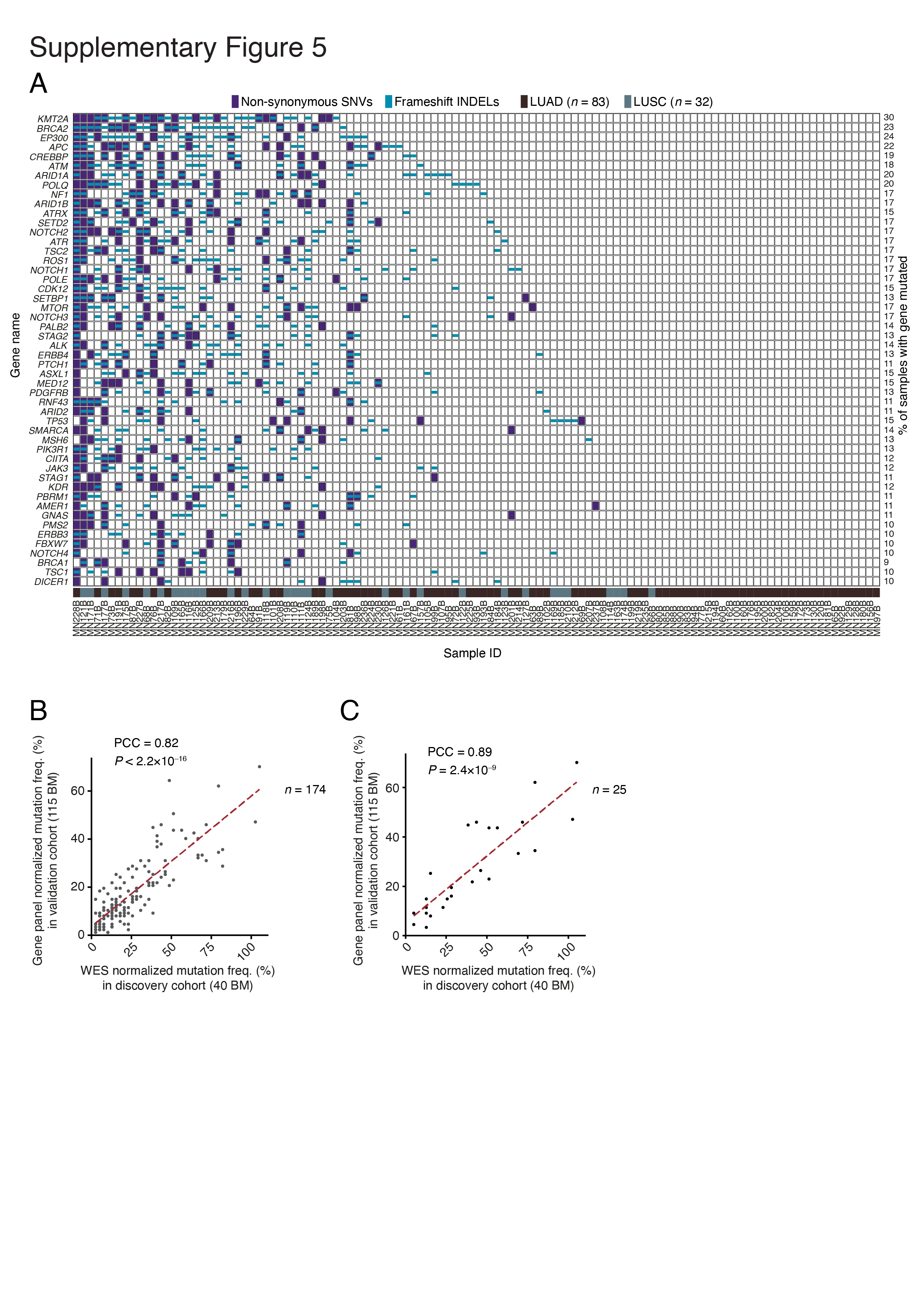
**

**Supplementary Fig. 5.** Validation of SNVs and indels in an independent cohort of BM samples. (**A**) Top-50 mutated genes in the 115 BM samples in the validation cohort profiled with the Glasgow Cancer Core Panel (see **Supplementary Table 9**). The sample IDs are the same as in **Supplementary Table 8**). (**B**) Correlation between the percentage of samples that have one of the genes in the Glasgow Cancer Core Panel (see **Supplementary Table 9**) mutated in the discovery vs. validation cohort. PCC, Pearson’s correlation coefficient. *P*, Wilcoxon test, two-tailed. Each dot represents one of the 174 genes (*n*) in the Glasgow Cancer Core Panel. Dashed red line, linear regression fit. (**C**) Same as in (B) but for 25 putative BM driving genes identified using CHASM (see **Methods**).

**2. Supplementary Tables**

Because of their size, all tables are provided as separate Excel files.

**Supplementary Table 1.** Main epidemiological and clinical features of the patients from which the 51 NSCLC-BM sample pairs in the discovery cohort were obtained.

**Supplementary Table 2.** Summary of the sequencing data presented in this study.

**Supplementary Table 3.** Percentage of the genome amplified (AMP) or deleted (DEL) in the 51 NSCLC-BM sample pairs in the discovery cohort.

**Supplementary Table 4.** Copy number of 87 cancer-associated genes assessed by Nanostring in 4 NSCLC-BM pairs and one additional BM sample in the discovery cohort.

**Supplementary Table 5.** Manually curated list comprising all COSMIC genes plus other relevant lung cancer associated genes listed in the IGV database.

**Supplementary Table 6.** List of putative NSCLC-BM driving genes identified in this study.

**Supplementary Table 7.** Characteristics of the 84 BM samples in the validation cohort profiled with CUTseq.

**Supplementary Table 8.** Characteristics of the 115 BM samples in the validation cohort profiled with the Glasgow Cancer Core Panel.
